# Overexpression of EGFR in Head and Neck Squamous Cell Carcinoma Is Associated with Inactivation of SH3GL2 and CDC25A Genes

**DOI:** 10.1371/journal.pone.0063440

**Published:** 2013-05-10

**Authors:** Guru Prasad Maiti, Pinaki Mondal, Nupur Mukherjee, Amlan Ghosh, Susmita Ghosh, Sanjib Dey, Jayanta Chakrabarty, Anup Roy, Jaydip Biswas, Susanta Roychoudhury, Chinmay Kumar Panda

**Affiliations:** 1 Department of Oncogene Regulation, Chittaranjan National Cancer Institute, Kolkata, India; 2 Cancer Biology and Inflammatory Disorder Division, CSIR-Indian Institute of Chemical Biology, Kolkata, India; 3 Department of Zoology, Presidency University, Kolkata, India; 4 Department of Pathology, Johns Hopkins School of Medicine, Baltimore, Maryland, United States of America; 5 Department of Surgical Oncology, Chittaranjan National Cancer Institute, Kolkata, India; 6 North Bengal Medical College, Sushruta Nagar,Darjeeling, West Bengal, India; Sapporo Medical University, Japan

## Abstract

The aim of this study is to understand the mechanism of *EGFR* overexpression in head and neck squamous cell carcinoma (HNSCC). For this reason, expression/mutation of *EGFR* were analyzed in 30 dysplastic head and neck lesions and 148 HNSCC samples of Indian patients along with 3 HNSCC cell lines. In addition, deletion/methylation/mutation/expression of *SH3GL2* (associated with *EGFR* degradation) and *CDC25A* (associated with dephosphorylation of *EGFR*) were analyzed in the same set of samples. Our study revealed high frequency of *EGFR* overexpression (66–84%), low frequency of gene amplification (10–32.5%) and absence of functional mutation in the dysplastic lesions and HNSCC samples. No correlation was found between protein overexpression and mRNA expression/gene amplification status of *EGFR*. On the other hand, frequent alterations (deletion/methylation) of *SH3GL2* (63–77%) and *CDC25A* (37–64%) were seen in the dysplastic and HNSCC samples. Two novel single nucleotide polymorphism (SNPs) were found in the promoter region of *SH3GL2*. Reduced expression of these genes showed concordance with their alterations. Overexpression of *EGFR* and *p-EGFR* were significantly associated with reduced expression and alterations of *SH3GL2* and *CDC25A* respectively. In-vitro demethylation experiment by 5-aza-2′-deoxycytidine (5-aza-dC) showed upregulation of *SH3GL2* and *CDC25A* and downregulation of *EGFR* expression in Hep2 cell line. Poor patient outcome was predicted in the cases with alterations of *SH3GL2* and *CDC25A* in presence of human papilloma virus (HPV) infection. Also, low *SH3GL2* and high *EGFR* expression was a predictor of poor patient survival. Thus, our data suggests that overexpression of *EGFR* due to its reduced degradation and dephosphorylation is needed for development of HNSCC.

## Introduction

Head and neck squamous cell carcinoma is the sixth most common cancer worldwide and it accounts for 30–40% of all cancer types in the Indian subcontinent [1]. Epidemiological studies have linked tobacco, betel-nut leaf quid, alcohol and infection with oncogenic type of HPV16/18 to the development of HNSCC [1]. In the progression model of HNSCC, number of alterations like overexpression of epidermal growth factor receptor (*EGFR*) protein, deletion in chromosome 9p21, p16/p14 inactivation, trisomy of chromosome 7 and telomerase activation were suggested to be associated with the development of hyperplastic lesions of head and neck [2]. Among these alterations, overexpression of *EGFR* is quite important due to its regulation of multiple cell signaling cascades. Nowadays, multiple therapeutic targets have been made against *EGFR* to treat this tumor, but success is still far behind [3].

Though, frequent (80–90%) overexpression of *EGFR* protein was seen in HNSCC yet amplification of this locus was not prevalent (10–30%) [4]. Even hemizygous of this locus showed overexpression of this protein [5], [6]. In addition, 1–7% of the tumor showed mutation of this gene [4]. This suggests that other mechanisms may be associated with the overexpression of *EGFR* in HNSCC. It was evident that binding of EGF to *EGFR* triggered a series of biochemical events including autophosphorylation of specific tyrosine residues (like Tyr 1045, Tyr 992, Tyr 975, Tyr 1068, Tyr 1173 etc) in its kinase domain [4] i.e activated EGFR protein level will be elevated without changing total EGFR protein level [7], [8]. The phosphorylation of Tyr-1045 is important as Cbl, an E3 ubiquitin ligase, interacts at this site for initiation of its endocytosis mechanism through CIN85-*SH3GL2* interaction [9]. Frequent deletion (29–37%), promoter hypermethylation (42–46%) and reduced expression (68%) of *SH3GL2* (located at chromosome 9p22.2) have been reported in head and lesions [1]. Its reduced expression has also been seen in laryngeal carcinoma [10]. It has mainly two domains. The SH3 domain of *SH3GL2* interacts with CIN85, whereas the LPAAT domain of SH3GL2 is associated with lysophophatydic acid acyl tyransferase activity which converts lysophosphotidic acid (LPA) into phosphatidic acid (PA) needed for membrane curvature for encapsulation of *EGFR* [9]. However, to the best of our knowledge, association of *SH3GL2* and *EGFR* in HNSCC has not yet been studied. On the other hand, Shang, et al [11] reported that *CDC25A*, a dual phosphatase, could regulate the activity of *EGFR* through dephosphorylation of the tyrosine residues. Its frequent deletion (53%) and reduced expression (64%) have been reported in HNSCC [1]. However, like *SH3GL2*, association of *CDC25A* with *EGFR* in HNSCC has not yet been studied.

Thus, to understand the molecular mechanism of *EGFR* protein overexpression, we analyze the alterations of *EGFR*, *SH3GL2* and *CDC25A* in the same set of HNSCC samples. At first, alterations (expression, gene amplification and mutation) of *EGFR* were analyzed in primary head and neck lesions of Indian patients and some HNSCC cell lines. Then, alterations (deletion/promoter methylation/mutation/expression) of SH3GL2 and *CDC25A* were analyzed in these samples. Our data suggests that overexpression of *EGFR* protein might be due to the impairment of the *SH3GL2* associated endocytosis mechanism, whereas down regulation of *CDC25A* in this tumor might lead to the *EGFR* protein in its active state.

## Materials and Methods

### Ethics Statement

The Institutional Ethical board of Chittaranjan National Cancer Institute, Kolkata approved the usage of Human specimens in this study. The above board approved usage of these human clinical samples specifically in this study, related to the involvement of the study of EGFR homeostasis in HNSCC. The tumor specimens were collected from the hospital section of Chittaranjan National Cancer Institute, Kolkata, after obtaining written, informed consent of the concerned patients, in stipulated format, approved by the above mentioned Institutional Ethical board of Chittaranjan National Cancer Institute, Kolkata, India. Blood samples were collected from healthy control with written informed consent as approved by the Institutional ethical Board.

### Patient Population, Tumor Tissues and Cell Lines

**Figure 1:**
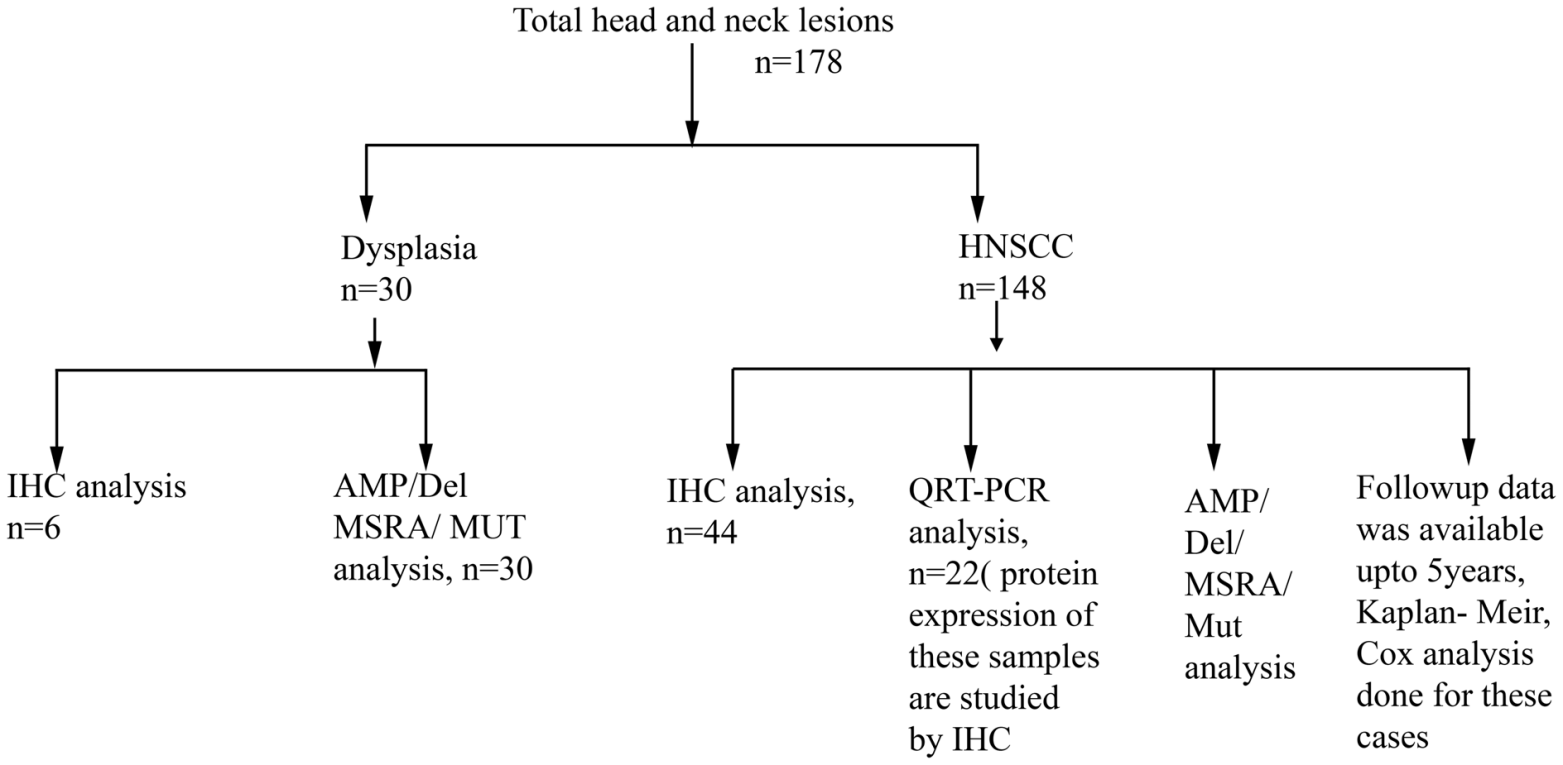
Tumor samples usage. Schematic representation of the usage of primary HNSCC tumor in different experimental analyses. N,sample number; HNSCC, head and neck squamous cell carcinoma; IHC, Immunohistochemical analysis; AMP, Gene amplification analysis; Del, deletion analysis; MSRA, Methylation Sensitive Restriction Analysis; Mut, Mutation analysis by SSCP; QRT-PCR, Real time-PCR quantification.

Total 178 tumor tissue samples of head and neck lesions as well as matched adjacent normal tissues were collected from Chittaranjan National Cancer Institute, Kolkata, India. The primary tumors were collected from patients after surgical resection having no previous treatment record. All these tumors were graded and staged according to UICC TNM classification. Samples were frozen immediately after collection at -80°C until use. Part of the freshly operated tissues was directly collected in TRIzol reagent (Invitrogen, San Diego, CA) for RNA isolation and another part was fixed in formalin and embedded in paraffin for immunohistochemical analysis. Clinicopathological information of the patients (n = 178) were presented in Table 1 and unrelated control (n = 52) having no previous and present history of HNSCC was presented in Table S1. Among HNSCC cell lines Hep2 and KB were procured from the National Centre for Cell Sciences, Pune, India and UPCI: SCC 084 was kindly provided by Prof. Susanne M. Gollin, University of Pittsburgh, PA. Summary of the total samples usage used in different experiment was shown in Figure 1.

**Table 1:** Clinicopathological feature of head and neck lesions.

	Clinical	No of samples	HPV 16/18 positivity		
	Features	(n = 178) (n%)	+ (n %)	− (n %)	p-value
**Age**		54.8±11.2	93(52.2)	85(47.8)	
	(Mean±SD)				
	Mean ≥54.8	84(47.2)	41(48.8)	43(51.2)	0.385
	Mean ≤54.8	94(52.8)	52(55.5)	42(44.5)	
**Primary sites**					
	BM	96(53.9)	51(53.1)	45(46.8)	0.156
	ALV	35(19.6)	10(28.6)	25(71.4)	
	TNG	27(15.1)	16(59.3)	11(40.7)	
	Others #	20(11.2)	15(75)	5(25)	
	Sex				
	Male	132(74.2)	68(51.5)	64(48.4)	0.741
	Female	46(25.8)	25(54.4)	21(45.6)	
**Tumor stage**					
	Dysplasia	30(16.8)	14(46.6)	16 (53.3)	
	TNM stage I	18(10.1)	5(27.8)	13(72.2)	
	TNM stage II	42(23.5)	27(64.3)	15(35.7)	0.214
	TNM stage III	48(26.9)	27(56.25)	21(43.75)	
	TNM stage IV	40(22.4)	22(55)	18(45)	
**Tumor**					
**Differentiation$**					
	WDSCC	84(47.2)	45(53.6)	39(46.4)	
	MDSCC	55(30.8)	32(58.2)	23(41.8)	
	PDSCC	9(5)	4(44.4)	5(55.6)	0.989
**Lymph node$**					
	Positive	42 (23.5)	25(59.5)	17(40.5)	0.462
	Negative	106 (59.5)	56(52.8)	50(47.2)	
**Tobacco**					
	Tobacco +	129 (72.4)	73(56.5)	56(43.4)	0.0341*
	Tobacco −	49(27.5)	19(38.7)	30(61.2)	

$ Excluding dysplasia,

# Samples including thyroid, nasopharyngs, larynx,

* Statistically significant.

**Abbreviation:** HPV, Human papiloma virus; SD, Standard deviation; BM, buccal mucosa; ALV, alveolus; TNG, tongue; TNM, tumor-node-metastasis; WDSCC, well differentiated squamous cell carcinoma; MDSCC, moderately differentiated squamous cell carcinoma; PDSCC poorly differentiated squamous cell carcinoma.

### Microdissection and DNA Extraction

The contaminant normal cells in the head and neck lesions were removed by microdissection procedure from cryosections (5 µm) using surgical knives under a dissecting microscope (Leica MZ16, Germany). The representative sections from different regions of the specimens were stained with hematoxylin and eosin for diagnosis as well as for marking of the dysplastic epithelium/tumor rich regions. The samples containing >60% dysplastic epithelium/tumor cells were taken for isolation of DNA according to the standard procedure [12]. High-molecular-weight DNA was extracted by proteinase-K digestion, followed by phenol/chloroform extraction [13].

### Immunohistochemical (IHC)/Immunocytochemical (ICC) Analysis

The protein expression of *EGFR*, *SH3GL2* and *CDC25A* was done by immunohistochemical analysis in 50 primary tumor samples and by immunoflorescence analysis in Hep2, KB, SCC084 according to the standard procedure as describe earlier [14]. The tissues were reacted overnight with primary antibodies *CDC25A* (sc-6947), *SH3GL2* (sc-10874), *EGFR* (sc-03) and *p-EGFR* (sc-57541) at a dilution of 1∶80 at 4°C. HRP conjugated rabbit anti-goat (sc-2020), goat anti rabbit (sc-2004) and goat anti mouse (sc-2005) secondary antibody was added 1∶500 dilutions. The slides were developed using 3–3′ diaminobenzidine (DAB) as the chromogen and counterstained with hematoxylin and photographed in Bright Field microscope (Leica DM1000, Germany). The staining intensity (1 = weak, 2 = moderate, 3 = strong) and the percentage of positive cells (<1 = 0, 1–20 = 1, 20–50 = 2, 50–80 = 3 and >80 = 4) were detected by 2 observers independently and by combining the two scores, final evaluation of expression was done (0–2 = low, 3–4 = intermediate, 5–7 = high) [6].

For immunofluroscence analysis, cover slip culture of Hep2, KB and SCC 084 cell lines were reacted with the same dilution of primary antibody of these genes after permeabilisation with 0.5% Triton X-100 and blocking with 5% BSA. After washing, the coverslips were incubated with FITC conjugated corresponding secondary antibody goat anti mouse (sc-2010), goat anti rabbit (sc-2012) and rabbit anti goat (sc-2777) at 1∶500 dilution and mounted with glycerol after thorough washing. Imaging of the cover slip was performed in florescence microscope (Leica DM4000 B, Germany).

### Quantification of *EGFR* Gene Copy Numbers

A quantitative measurement of *EGFR* amplification was carried out using differential polymerase chain reaction (DPCR) method [15] using the primer as shown in Table S2. The primer from exon-20 of *EGFR* gene was selected for amplification analysis. The dopamine D2 receptor gene (DRD2) gene was used as internal control due to low frequency of alterations reported in HNSCC [15]. The gene amplification by DPCR method is validated by real-time quantitation. See Supplementary method for details of the protocol (Doc S1).

### Mutation Analysis

The mutation of *EGFR* and *SH3GL2* was screened in 30 dysplastic lesions, 148 invasive samples and 3 oral cancer cell lines by single strand conformation polymorphism (SSCP) analysis using [α-P32] dCTP as described by Tripathi el al [16].The mutational hotspot region of kinase domain encompassing the exon-18 to exon-21 were selected for mutation analysis of *EGFR*. For mutation analysis of *SH3GL2*, important enzymatic domain and alternating splice site of the gene were selected. Exon 9 & exon-10 encoded the SH3domain and exon-1 & exon-2 encoded the lysophosphatydic acid acyl transferase enzymatic domain of the gene. The alternating splice sites were located in exons 3, 4, &5. All these exons were selected for mutation analysis of *SH3GL2*. Primer sequences and locations were presented in Table S2 & Figure S2b, A.4a. Electrophoresis was done in 6% nondenaturing polyacrylamide gel with 10% glycerol at 2 W for overnight and autoradiographed on X-ray film (Kodak, USA). Sequencing of both strands of samples showing abnormal band shift was done using 3130xl-Genetic Analyzer (Applied Biosystems, USA).

### Deletion Analysis of *SH3GL2* and *CDC25A*

In microsatellite based deletion mapping, a standard polymerase chain reaction (PCR) containing [γ-P32] ATP end labeled forward primer was done in a 20 µl reaction volume according standard procedure as describe earlier [14]. The microsatellite markers are selected on the basis of their map positions and heterozygosity (http://www.ensembl.org, Release-49). An intragenic microsatellite marker D9S157 located 17.61 Mb from p-ter of chr. 9 was taken for analysis of *SH3GL2* and D3S3560 locus located 48.16 Mb from p-ter of chr. 3 and 4.8 Kb telomeric of the gene was taken for deletion analysis of *CDC25A*. Details of the markers was shown in Table S3.

### Promoter Methylation Analysis

The promoter methylation of the candidate genes *SH3GL2* and *CDC25A* was analyzed by PCR-based methylation sensitive restriction analysis (MSRA) method using MspI/HpaII restriction enzymes (Sibenzyme, Russia) using standard procedure as describe previously [14]. Details of primer sequences are listed in Table S2. The methylation data obtained by MSRA was validated by methylation-specific PCR (MSP) according to standard procedure [17]. See Supplementary method for details of the protocol (Doc S1).

### mRNA Expression Analysis

mRNA expression of *SH3GL2*, *CDC25A* and *EGFR* were analyzed in 22 primary HNSCC samples and their corresponding normal head and neck tissues and 3 cell lines (Hep2, KB and SCC 084) using primers as mentioned in Table S2. Total RNA was isolated from samples using TRIzol reagent according to the manufacturer’s protocol (Invitrogen). Reverse transcription was performed with 1 µg total RNA using Random hexamer (Invitrogen) and M-MuLV-Reverse Transcriptase (Promega, USA). Relative expression of the gene was measured by Real-Time quantitation method as described previously [14]. β2-microglobulin (B2M) was used as a internal control.

### Cell Lines and 5-aza-dC Treatment

In our previous analysis of 5-aza-dc experiment at different concentrations (5–50 µM) it was evident that the cell viability was unaltered up to 5 days of 5-aza-dc treatment at 20 µM concentration. For this reason, the 5-aza-dc concentration was taken upto 20 µM in Hep2 cell line. Subconfluent cultures of Hep2 was treated with 5 M, 10 M and 20 M demethylating agent, 5-aza-dC (Sigma, USA) for 5 days. Controls without 5-aza-dC were cultured concomitantly in the same manner. After completion of treatment, cells are harvested and proceeded for next experiment.

For kinetic study, subconfluent cultures of Hep2 cell line were treated with 20 µm 5-aza-dc in six Petri dish and the cells were harvested at different time point like 0 hour, 24 hours, 48 hours, 72 hours, 96 hours and 120 hours after aza treatment and protein was isolated from the cells. For immunoflorescence analysis after aza treatment, Hep2 cells were grown over night on cover slip and treated with 20 µm 5-aza-dc for 72 hour. Then the cells were fixed with chilled methanol and used for immunoflorescence analysis.

### Gene Knock Down by siRNA Treatment

The siRNA mediated knock down of SH3GL2 and CDC25A experiment was done in oral cancer cell line SCC084 according to standard procedure [18]. Briefly, about 2×10^5^ cells were plated in 35 mm petri dish. After 24 hour, media was replaced with serum and antibiotic free media and siRNAs were transfected using Lipofectamin 2000 (Invitrogen) according to the manufacturer protocol. The siRNA of SH3GL2 (SC-35304), CDC25A (SC-29254) and scrambled control (SC-37007) were purchased from Santa Cruz Biotechnology, CA, USA and were used at a final concentration of 80 pmole. After siRNA transfection, cells were harvested at different time point like 24 hour, 48 hour, 72 hour and 96 hour for each gene and RNAs were isolated to find out the time point for maximum gene knock down by real time quantitation. After characterizing the time for optimum gene knock down, cells were again treated with siRNA and harvested after particular time to isolate the protein.

### Western Blot Analysis

Proteins were extracted from cells by using standard protocol [19] and transferred to PVDF membrane after gel electrophoresis. The PVDF membrane was incubated with primary antibody for *EGFR*, *p-EGFR*(sc-12351), *SH3GL2*, *CDC25A,* Actin (sc-8432) and alpha-tubulin (sc-5286) and HRP-tagged secondary antibodies and developed with luminol (sc-2048; Santa Cruz Biotechnology, USA). The signal intensities were scanned by densitometric scanning (Bio-Rad GS-800). The same membrane was used for incubation with different antibodies after stripping with 0.2M NaOH (See Supplementary method for details of the protocol Doc S1).

### Detection of HPV-16 and HPV-18

Presence of HPV in the head and neck lesions was detected by PCR using primers (MY09 and MY11) from the consensus L1 region followed by typing of HPV 16/18 in the L1 positive samples as described previously [14].

### Statistical Analysis

Fisher’s exact test was used to determine the association between tumors genetic profile and different clinicopathological features. All statistical tests were 2-sided and considered significant at probability value, p<0.05. Survival curves were calculated according to Kaplan–Meier method in 148 HNSCC samples. For this method, p values were evaluated by the log rank test for censored survival data. A Cox-proportional hazards regression model was used to test the statistical significance of several potential prognostic factors like clinical stage, tumor site, tobacco exposure, HPV infection and alterations of the candidate TSGs that were jointly predictive of overall survival of the patients. From this model we estimated the hazard ratio (HR) for each potential prognostic factor with a 95% confidence interval (CI) in univariate and a multivariate fashion. All the statistical analysis was performed using statistical programs Epi Info 6.04 b, SPSS 9.0 (SPSS, Chicago, IL).

### Bioinformatics Analysis

All the oligonucleotides primers used in different experiment were designed using primer-3 software 20. Transcription factor binding site of SH3GL2 promoter was analyzed uising on line server Alibaba 2.1 TF Binding Prediction software 21.

## Results

### Molecular Alterations of *EGFR*

#### Immunohistochemical/immunocytochemical analysis

**Figure 2:**
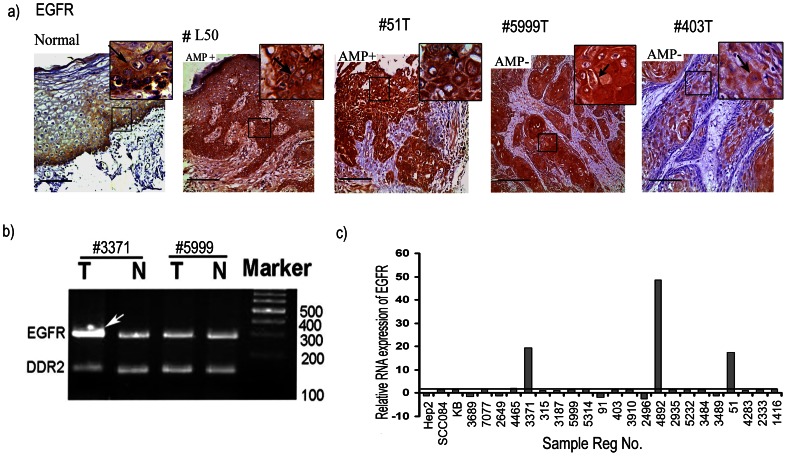
Molecular alterations of EGFR in HNSCC. a) Immunohistochemical analysis of EGFR proteins in normal, dysplasia and HNSCC. Distinct cytosolic/membrane expression of EGFR in the basal lining/parabasal cells/spinous cells of normal oral epithelium, dysplasia and HNSCC samples. EGFR has high cytoplasmic expression in the basal cells followed by gradual decrease in parabasal and spinus layer of normal oral epithelium. #L50 (AMP+), #51T(AMP+) and #5999T(AMP-) showed overexpression of cytoplasmic and membrane EGFR. # 403 (AMP-) showed medium EGFR expressions. Arrows point to membrane/cytoplasmic expression. Magnification of tissue samples is 20×, and inset magnification is 40×. Scale bars in tissue sections represent 100 µm. AMP+/−, gene amplification present/absent. b) Representative gel diagram featuring amplification of EGFR locus. DDR2 locus was used as the control locus. The sample number was indicated above the figure. Arrow head indicate the amplified band in #3371T. T: Tumor DNA, N: corresponding normal DNA. c) Quantitative RT-PCR showing mRNA expression pattern of EGFR in HNSCC samples and cell lines. Bars represent the gene expression normalized to β2-microglobulin and relative to corresponding adjacent normal tissues, using 2-ddCt method. The line illustrates the mean fold of expression level. X-axis indicates the sample numbers.

In normal oral epithelium, high cytoplasmic as well as membrane bound expression of *EGFR* was seen in basal layer followed by gradual decrease in parabasal and spinous layers (Figure 2a). In premalignant oral lesions, high cytoplasmic as well as membrane bound expression of *EGFR* was seen in basal, parabasal and part of the spinous layer in 66% (4/6) of dysplastic lesions. In invasive HNSCC tumor, overexpression of *EGFR* was observed in 84% (37/44) samples irrespective of tumor stages (Figure 2a). In immunocytochemical analysis of HNSCC cell lines, high expression of *EGFR* was seen in the following order: Hep2> KB>SCC084 (Figure S3). This indicates that high expression of *EGFR* is needed for development of dysplastic lesions and subsequent stages of HNSCC.

#### Gene amplification analysis

In DPCR analysis, about 26.4% (47/178) of the head and neck lesions showed *EGFR* amplification (Figure 2b). Gradual increase in amplification has been seen from dysplasia (10%, 3/30) to invasive lesions (31%, 46/148) (Figure S1a). No amplification was found in the HNSCC cell lines. The DPCR analysis showed concordance (p = 0.0007) with gene amplification analysis through real time PCR method (Table S4). Thus, *EGFR* overexpression through gene amplification might not be the sole mechanism.

#### Mutation analysis

In SSCP analysis, about 13% (24/178) and 20% (35/178) of the HNSCC samples showed altered bands in exon-18 and exon-20 respectively (Table S5a & Figure S1b, c, d). No altered band was observed in exon-19 and exon-21. Also, no altered band was found in the three cell lines. In sequencing analysis of the samples having altered bands it was evident that a SNP (G>A: rs17337107) in the down stream of exon-18 i.e. intron-18 and a SNP (G>A: rs1050171) at codon 787 in exon- 20 were located (Figure S1b, e & Table S5c). However, 12/24 of tumor samples showed A>G transition mutation in the SNP (G>A: rs1050171) (Table S5b). Thus, functional mutation in *EGFR* is a rare event in HNSCC.

#### mRNA expression analysis

It was revealed that 12% (3/25) of the HNSCC samples showed overexpression of *EGFR* mRNA with mean fold expression of 1.46 (±10.58) (Figure 2c). The mRNA expression showed concordance with gene amplification (p = 0.0025), while no correlation between mRNA expression and protein expression was observed (p = 0.5528) (Table 2). No overexpression was seen in the three HNSCC cell lines. This suggests that overexpression of *EGFR* protein is not associated with its mRNA expression.

**Table 2:** Correlation of molecular alterations with RNA/protein expression of the genes SH3GL2 and CDC25A and the relationship between the protein expression of EGFR vs SH3GL2 and CDC25A vs p-EGFR.

	EGFR					SH3GL2				CDC25A					p-EGFR^1045^
	Genetic	Expression				Genetic	Expression			Genetic		Expression			Expression
	Altn	RNA		Protein		Altn	RNA		Protein	Altn		RNA		Protein	Protein
**Dysplastic lesions**															
L7	Amp−	ND		++		D−M−	ND		+++	D−		ND		++	++
L48	Amp−	ND		+++		D−M+	ND		++	D−		ND		+++	+
L50	Amp+	ND		+++		D−M+	ND		++	D−		ND		++	++
L52	Amp−	ND		++		D+M−	ND		+	D−		ND		+++	++
L58	Amp−	ND		+++		D−M+	ND		+	D−		ND		+++	+
L127	Amp−	ND		+++		D+M+	ND		+	D+		ND		+	+++
**HNSCC cell line**															
Hep2	Amp−	Normal		+++		D−M+	↓		+	D−		↓		++	++
SCC084	Amp−	Normal		+		D−M−	Normal		+++	D−		Normal		+++	++
KB	Amp−	Normal		++		D−M−	Normal		+++	D−		Normal		+++	++
**HNSCC samples**															
3689	Amp−	Normal		++		D−M+	↓		++	D−		Normal		++	++
7077	Amp−	Normal		+++		D−M+	↓↓		++	D+		↓↓		+	+++
2649	Amp−	Normal		+++		D+M−	↓		+	D+		↓↓		+	+++
4465	Amp−	Normal		+++		D−M+	↓		++	D−		Normal		+++	+
3371	Amp+	↑		+++		D−M+	↓		++	D−		↓		+++	+
315	Amp−	Normal		+++		D+M−	↓↓		+	D−		Normal		++	++
3187	Amp−	Normal		+++		D+M+	↓↓		+	D−		Normal		+++	+
5999	Amp−	Normal		+++		D+M−	↓		+	D+		↓↓		+	+++
5314	Amp−	Normal		++		D−M+	↓↓		++	D+		↓↓		+	+++
91	Amp−	Normal		+++		D−M+	↓		++	D−		Normal		++	++
403	Amp−	Normal		+++		D+M+	↓↓		++	D−		Normal		+++	+
3910	Amp−	Normal		+++		D−M+	↓		++	D−		↓		++	++
2496	Amp−	Normal		+++		D+M+	↓↓		+	D+		↓↓		+	+++
4892	Amp+	↑		+++		D+M−	↓↓		+	D−		↓		++	++
2935	Amp−	Normal		+++		D−M+	↓		++	D−		↓		++	++
5232	Amp−	Normal		+++		D+M+	↓↓		+	D+		↓		+	+++
3484	Amp−	Normal		+++		D+M+	↓↓		+	D−		Normal		+++	+
3489	Amp−	Normal		+++		D−M+	↓		++	D−		↓		++	++
51	Amp+	↑		+++		D+M+	↓↓		+	D−		Normal		++	++
4283	Amp−	Normal		+++		D+M−	↓↓		+	D+		↓↓		+	+++
2333	Amp−	Normal		++		D−M−	Normal		+++	D+				+	+++
1416	Amp−	Normal		+++		D+M−	↓↓		+	D+		↓↓		+	+++
p value	0.0025*		0.5528		0.00043*			0.00003*			0.0027*		0.011*		0.00005*
				0.0011*									0.0117*		

D+/−: Deletion (MA, LOH) positive/negative, M+/−: Methylation positive/negative,/: increased/decreased gene expression compared to normal. ND: Fresh tissues were unavailable for RNA isolation, *: statistically significant (p<0.05).

### Molecular Alterations of *SH3GL2* and *CDC25A*

#### Deletion analysis

**Figure 3:**
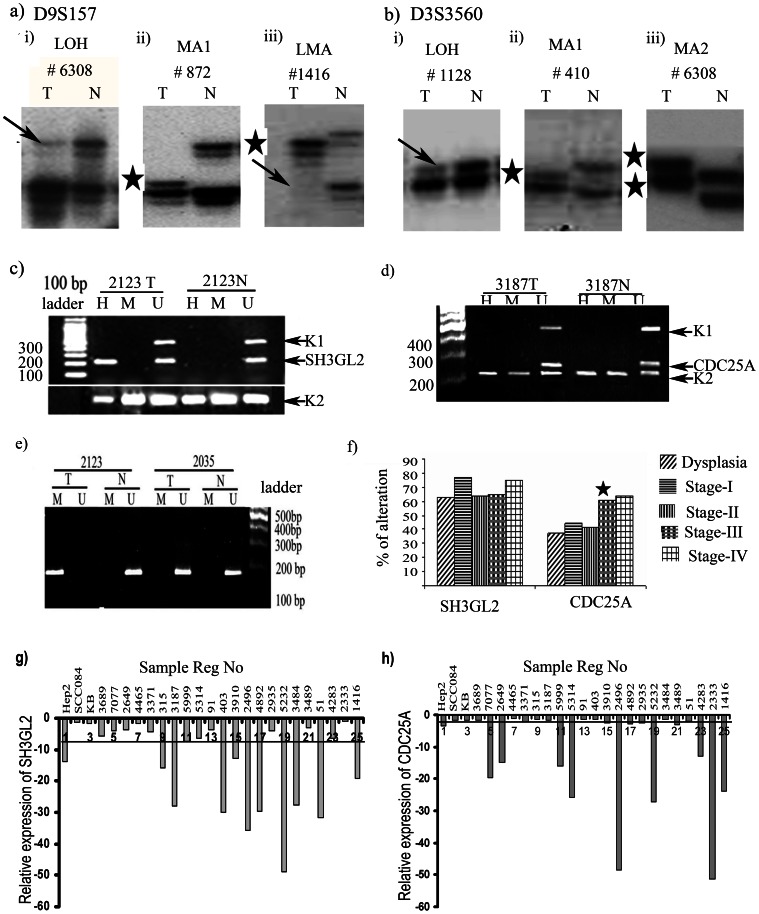
Molecular alterations of SH3GL2 and CDC25A. a) Representative autoradiographs showing deletion and MA in HNSCC samples at D9S157 marker loci. (i) LOH, loss of heterozygosity, (ii) MA-I, microsatellite size alteration of one allele, (iii) LMA microsatellite size alteration of of one allele and LOH in other allele. b) Genetic alterations of CDC25A analyzed by microsatellite based deletion mapping showing deletion and MA in HNSCC at D3S3560 marker loci (i) LOH loss of heterozygosity, (ii) MA-I microsatellite size alteration of one allele. (iii) MA2 microsatellite size alteration of both alleles. Arrow heads indicate the lost allele and star indicates the allele with MA. Representative gel electrophorogram showing the methylation status of c) SH3GL2 and d) CDC25A in tumor samples and in corresponding normal sample by MSRA. SH3GL2 showed methylation in tumor sample and absence of methylation in adjacent normal samples. Promoter methylation of CDC25A was absent in both tumor and normal samples. h, amplicon obtained with primer for HpaII digested DNA; m, amplicon obtained with primer for MspI digested DNA; u, amplicon obtained with primer for undigested DNA. e) The methylation analysis by MSRA was validated by MSP after bisulphate modification of DNA. The sample #2123T showed the methylalaton specific PCR band, but #2123N and #2935TN showed unmethylation specific PCR band. U; amplicon obtained with primer for modified unmethylated DNA, M amplicon obtained with primer for modified methylated DNA, T tumor DNA, N corresponding normal tissue DNA. f) Frequency of overall alterations of the genes SH3GL2 and CDC25A observed in dysplasia and different stages of HNSCC samples. Significant increase in alteration with tumor progression is shown by asterisk. Q-RT PCR showing reduced expression of g) SH3GL2 and h) CDC25A in HNSCC tumor. Bars represented the gene expression normalized to β2-microglobulin and relative to adjacent normal tissues using the 2?^-ddCt^ method. The line illustrates the mean decreased level of the genes. X-axis indicates samples.

After excluding non-informative tumor samples (21 of 178 at *SH3GL2* and 60 of 178 at *CDC25A* locus), high frequency of deletion was observed in *SH3GL2* (34%, 54/157) and *CDC25A* (52%, 56/108) loci (Figure 3a, b). The deletion frequencies in *SH3GL2* and *CDC25A* were observed in 27% (7/26) and 37% (6/16) dysplastic lesions respectively. Comparable frequency of *SH3GL2* deletion (30–46%) was observed in subsequent stages, while deletion frequencies of *CDC25A* (37–41%) were comparable upto stage-I/II followed by significant increase (61–64%) in stage-III/IV of the HNSCC samples (Figure 3f, Figure S4). In addition, MA was observed in both *SH3GL2* and *CDC25A* loci in varying frequencies (4–16%) (Figure S4). This indicates that deletion might be one of the mechanisms of inactivation of *SH3GL2* and *CDC25A* in HNSCC, as reported in our earlier studies [1], [14]. A statistically significant correlation was observed between *SH3GL2* and *CDC25A* alterations (p = 0.05) in tumor samples (Table S6).

#### Promoter methylation analysis

In MSRA, high frequency (44%, 78/178) of promoter methylation was seen in the *SH3GL2* gene in HNSCC lesions (Figure 3c). No promoter methylation of *CDC25A* was observed in the samples as seen in our previous report (Figure 3d) [14]. In the HNSCC cell lines, Hep2 showed methylated promoter of *SH3GL2*, whereas no methylation was detected in SCC084 and KB. The results were validated in randomly chosen 30 HNSCC samples and 3 oral cancer cell lines using MSP method after bisulfite modification of DNA (Figure 3e). Concordance was seen between MSRA and MSP analysis (p = 0.009) (Table 3). In dysplastic lesions 40% (12/30) of the samples showed promoter methylation of *SH3GL2* with comparable frequency (43–46%) of methylation in subsequent stages of HNSCC (Figure S4a). Thus it was evident that overall alterations (deletion/methylation) of *SH3GL2* was high (63%, 19/30) in dysplastic lesions and became comparable (64%–77%) in subsequent stages of tumor progression (Figure 3f).

**Table 3:** Correlation of the two methods of promoter methylation analysis.

Sample		SH3GL2	Sample		SH3GL2
4446	MSRA	+	3187	MSRA	+
	MSP	+		MSP	+
3371	MSRA	+	4145	MSRA	−
	MSP	+		MSP	−
315	MSRA	−	3484	MSRA	+
	MSP	−		MSP	+
5314	MSRA	+	363	MSRA	−
	MSP	+		MSP	−
91	MSRA	+	3689	MSRA	+
	MSP	−		MSP	+
5138	MSRA	+	206	MSRA	−
	MSP	+		MSP	−
2935	MSRA	−	1108	MSRA	+
	MSP	+		MSP	+
4892	MSRA	−	5314	MSRA	+
	MSP	−		MSP	+
3910	MSRA	+	2507	MSRA	−
	MSP	+		MSP	+
3127	MSRA	−	2323	MSRA	+
	MSP	−		MSP	−
2496	MSRA	−	410	MSRA	+
	MSP	+		MSP	+
4283	MSRA	−	7216	MSRA	+
	MSP	−		MSP	+
5232	MSRA	+	558	MSRA	−
	MSP	+		MSP	−
2649	MSRA	−	1332	MSRA	+
	MSP	−		MSP	+
3006	MSRA	−	% of	MSRA	57% (17/30)
	MSP	+	methylation	MSP	63% (19/30)
L50	MSRA	+	Significance	p value	0.00009*
	MSP	+	level		

MSRA, methylation sensitive restriction enzyme assay; MSP, Methylation specific PCR.;

* statistically significant; +, Methylation positive; −, Methylation negative.

#### Mutation analysis of *SH3GL2*

In SSCP analysis, altered bands in exon-1 were seen in 18% (32/178) HNSCC samples and also in their respective normal samples (Figure S2b & Table S7a). No other exons showed any altered band. In sequencing analysis of the samples having altered bands in exon-1, two novel sequence variations at −31 (C>T) and −64 (G>T) flanked by two reported SNPs (−4 G>C, rs4961573; −86 C>G, rs11540996) were found in 2/32 and 10/32 tumor and respective normal samples respectively (Figure S2c).

To identify the nature of observed sequence variations, sequencing analysis of these variants was done in 52 control DNA samples. The −31 heterozygous allele variant (C>T) was observed in 4% (2/52) control samples (Table S7b, c). On the other hand, 8% (4/52) control samples were heterozygous with G>T variation at −64 nucleotide position. This suggests the presence of two novel SNPs in the −31 and −64 nucleotide positions. Both the sequence variation was submitted in the gene bank (accession no. rs112820965 & rs201266191). Interestingly, the frequency of T allele at −64 G>T variation seems to be higher (0.16) in HNSCC patients than the control population (0.04), whereas no such preference has been seen in the C>T variation in the −31 nucleotide position (Table S7b, c). A GC Factor (GCF) transcription binding site was identified at −64 nucleotide position by Insilco analysis using Alibaba 2.1 TF binding prediction online software (Figure S2d). However, large number should be analyzed to find their association in HNSCC. Thus, it suggests that mutation of *SH3GL2* is a rare event in HNSCC.

#### mRNA expression analysis of *SH3GL2* and *CDC25A*

It was evident that 80% (20/25) primary HNSCC samples showed ≥2 fold reduction of *SH3GL2* mRNA expression with 6.4 (±13.5632) mean fold reduction (Figure 3g). In case of *CDC25A* reduced expression was seen in 60% (15/25) of the samples with 2.4 (±14.41543) mean fold reduction (Figure 3h). No change in expression pattern of genes was seen in the cell lines except reduced expression of *SH3GL2* and *CDC25A* in Hep2. The reduced expression of *SH3GL2* and *CDC25A* in HNSCC showed concordance with their genetic alterations (deletion/methylation) (p = 0.00043; p = 0.00278) (Table 2).

#### Protein expression analysis of *SH3GL2*, *CDC25A* and *p-EGFR*

**Figure 4:**
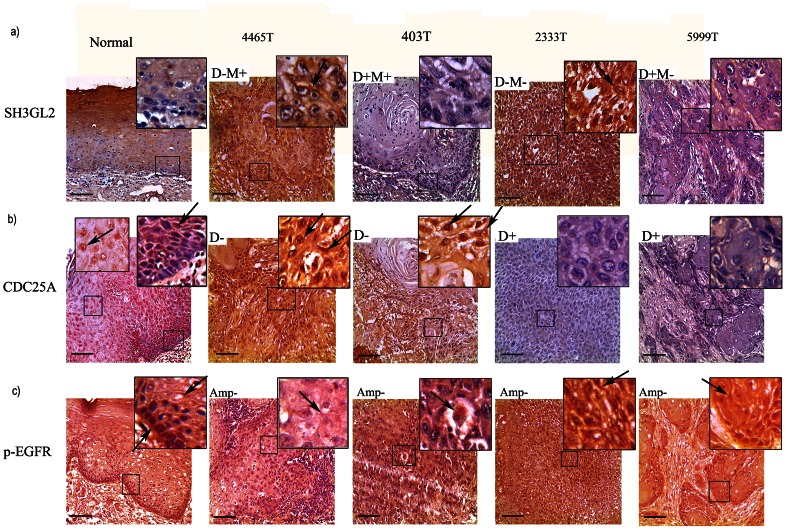
Immunohistochemical analysis of SH3GL2, CDC25A and p-EGFR. a) Distinct cytoplasmic/membrane expression of SH3GL2 in the basal lining/parabasal cells of normal oral epithelium and primary HNSCC samples were seen. Spinus layer showed high expression of the gene. #403T and #5999T showed low expression, #4465T and #2333T showed intermediate/high expression expression level of SH3GL2. b) Differential nuclear and cytoplasmic expression of CDC25A was seen in basal/parabasal/spinus layer cells of normal oral epithelium and tumor samples. Low cytoplasmic and nuclear expression of CDC25A was evident in basal and parabasal cells of normal epithelium, but higher expression in spinus layer. #2333T and #5999T showed low expression, #4465T and #403T showed high/intermediate nuclear and cytoplasmic expression level of CDC25A. Arrows pointed to nuclear/cytoplasmic/membrane expression. c) Distinct cytoplasmic and membrane bound expression of p-EGFR was seen in normal oral epithelium. The expression was high in basal layer but gradually decreased in parabasal and spinus layer. Arrows pointed to cytoplasmic/membrane/nucleus expression. #2333T and #5999T showed high expression, #4465T and #403 showed reduced expression of p-EGFR. Magnification of tissue samples is 20X, and for inset in tissues magnification is 40X. Scale bars in tissue sections represent 100 µm.

Immunohistochemical analysis revealed a differential expression pattern of *SH3GL2*, *CDC25A* and *p-EGFR* in the basal/parabasal/spinous cells of normal oral epithelium (Figure 4a, b, c). *SH3GL2* showed a diffused low cytoplasmic expression in the basal/parabasal cells and high cytoplasmic/membrane expression in spinous cells of normal oral epithelium. Low and diffuse nuclear and cytoplasmic expression of *CDC25A* was seen in basal and parabasal layers of normal oral epithelium followed by high nuclear expression in the spinous layers. Like *EGFR*, high nuclear and cytoplasmic expression of *p-EGFR* seen in basal layer gradually decreased in parabasal and spinus layers (Figure 4c). It seems that reduced expression of *SH3GL2* and *CDC25A* in normal basal oral epithelium might lead to high expression of *EGFR* and *p-EGFR* essential for maintenance of basal stem cell proliferation.

In primary tumors, reduced expression of *SH3GL2* was evident in 78% (39/50) of the samples (Figure 4b). Mainly cytoplasmic expression of *SH3GL2* was seen in KB and SCC084 with reduced expression in Hep2 (Figure S3b, c, a). Concordance was seen between reduced expression of *SH3GL2* and its molecular alterations in the samples (p = 0.00003) (Table 2). In addition, reduced expression of *SH3GL2* showed significant association (p = 0.0011) with overexpression of *EGFR* in the samples (Table 2). In the tumors, low or intermediate level of nuclear and cytoplasmic expression of *CDC25A* was observed in 70% (35/50) of the samples. Cytoplasmic and perinuclear expression of *CDC25A* was seen in the cell lines with low expression in Hep2 (Figure S3a, b, c). The expression of *CDC25A* was concordant with its deletion pattern of the samples (p = 0.011) (Table 2). In case of *p-EGFR* ^,^ high/intermediate nuclear and cytoplasmic expression was seen in 82% (41/50) of the samples (Figure 4c). Cytoplasmic and nuclear expression of *p-EGFR* was seen in the cell lines (Figure S3a, b, c). Interestingly, the high expression of *p-EGFR* in the tumors showed correlation with reduced expression of *CDC25A* (p = 0.0117 ) (Figure 4b, c and Table 2).

### Validation of *SH3GL2* and *CDC25A* Mediated *EGFR* Regulation

**Figure 5:**
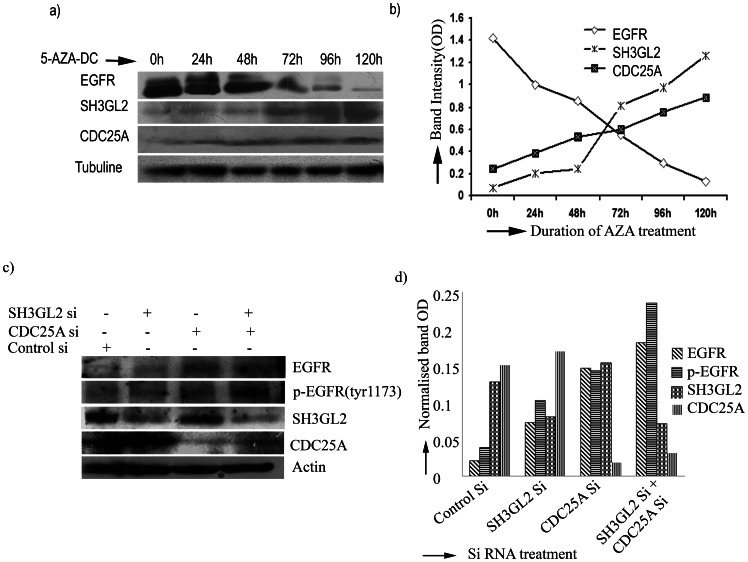
SH3GL2 and CDC25A mediated EGFR homeostasis. **a)**EGFR was degraded due to upregulation of SH3GL2 and CDC25A by 5-aza-dc treatment. Hep2 cell line was incubated with 20 µm of 5-aza-dc up to 120 h. Cells were harvested after zero hour of treatment and then every 24 h interval. Equal amounts of protein were subjected to western blotting. The amount of EGFR protein decreased gradually and degradation was maximum after 120 hour. Similarly, the expression of SH3GL2 and CDC25A was gradually increased after treatment. b) The amount of proteins (normalized band OD) was plotted as a function of time of 5-aza-dc treatment. The intensity of the bands were determined by densitometry and normalized with tubulin. c) SCC084 cell line was treated with siRNA of CDC25A and SH3GL2. Protein expression of the genes were analysed by western blot. Expression of EGFR and phosphorylated EGFR were assayed during knock down either of SH3GL2 and CDC25A or of both. Both EGFR and p-EGFR level was up regulated due to reduction of SH3GL2 and CDC25A. d) The amount of proteins (normalized band OD) was plotted. The intensity of the bands were determined by densitometry and normalized with actin. The bar diagram showing the level of EGFR and p-EGFR up regulation during siRNA treatment of SH3GL2 and CDC25A.

**Figure 6:**
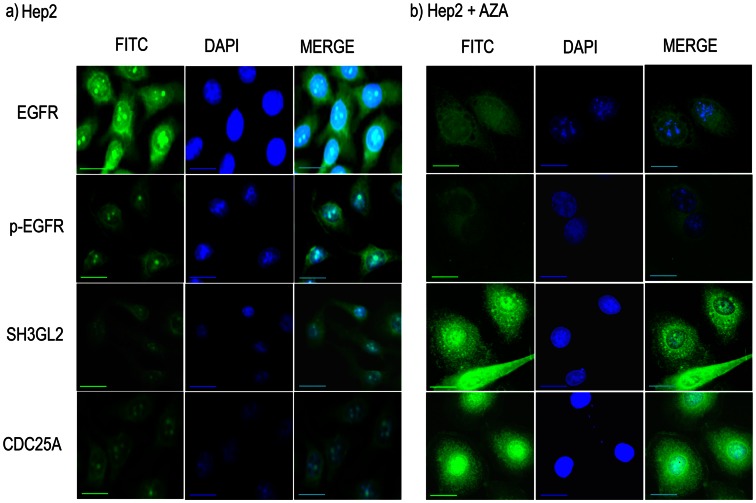
Immunocytochemical analysis of EGFR, SH3GL2, CDC25A and p-EGFR. over night subconfluent cover slip culture of Hep2 cell line was treated with 5-aza-dc for 72 hour and protein expression of the genes were analyzed by ICC after fixing the cells. EGFR and p-EGFR showed reduced expression in treated cells (b) compare to non treated cells (a). On the contrary, SH3GL2 and CDC25A showed upregulation of cytoplasmic/nuclear expression after aza treatment (b) compare to untreated cells (a). Scale bars in microphotograph represent 50 µm.

To find out the *SH3GL2* and *CDC25A* mediated regulation of *EGFR*/*p-EGFR* demethylation experiment in presence of 5-aza-dc was done in Hep2 cell line. It was evident that the mRNA expression of *SH3GL2* and *CDC25A* was gradually increased with increasing concentration of 5-aza-dc with significant increase of *SH3GL2* at higher concentration (Figure S5). Interestingly, at 20 µM concentration of 5-aza-dc *EGFR* protein expression was gradually decreased with significantly low after 48 hours of treatment and became considerably low at 120 hours (Figure 5a, b). On the other hand, *SH3GL2* protein expression was significantly increased after 48 hours of treatment and became high at 120 hours. Similarly, gradual increase in *CDC25A* protein expression was seen with time of treatment. In ICC analysis of expression of these proteins in Hep2 after treatment with 20 µm 5-aza-dc for 72 hours, high expression of *SH3GL2* and *CDC25A* were seen in nucleus and cytoplasm, whereas expression of both *EGFR* and *p-EGFR* were considerably reduced (Figure 6). The percent of increase or decrease of the proteins was shown in Table S8.

To confirm the SH3GL2 and CDC25A mediated regulation of EGFR/p-EGFR, further siRNA mediated knockdown experiments of SH3GL2 and CDC25A were performed in SCC084 cell line. The mRNA expression of SH3GL2 and CDC25A was analyzed after siRNA transfection at 24 hour, 48 hour, 72 hour and 96 hour. Maximum reduction in expression of the genes was seen after 72 hour of siRNA treatment (data not shown). In concordance with mRNA expression, protein expression of SH3GL2 and CDC25A was significantly reduced after 72 hour of siRNA treatment (Figure 5 c &d). Interestingly, EGFR and p-EGFR expression were up regulated after knock down of either SH3GL2 or CDC25A. Moreover, simultaneous knock down of SH3GL2 and CDC25A resulted significant up regulation of both EGFR and p-EGFR (Figure 5 c &d).

### Association of HPV with Alterations of *SH3GL2*, *CDC25A* and *EGFR*

Infection by HPV is considered as one of the important etiological factors for HNSCC development. HPV typing was done in this study to analyze the frequency of high-risk HPVs in our samples. HPV DNA was detected in 52.2% (93/178) of the tumors. Among the HPV positive samples, 92.5% (86/93) were HPV-16-positive and 7.5% (7/93) were HPV-18 positive. HPV infection was found to be significantly associated with tobacco consumption (p = 0.0341) (Table 1).

### Clinicopathological Correlation

**Figure 7:**
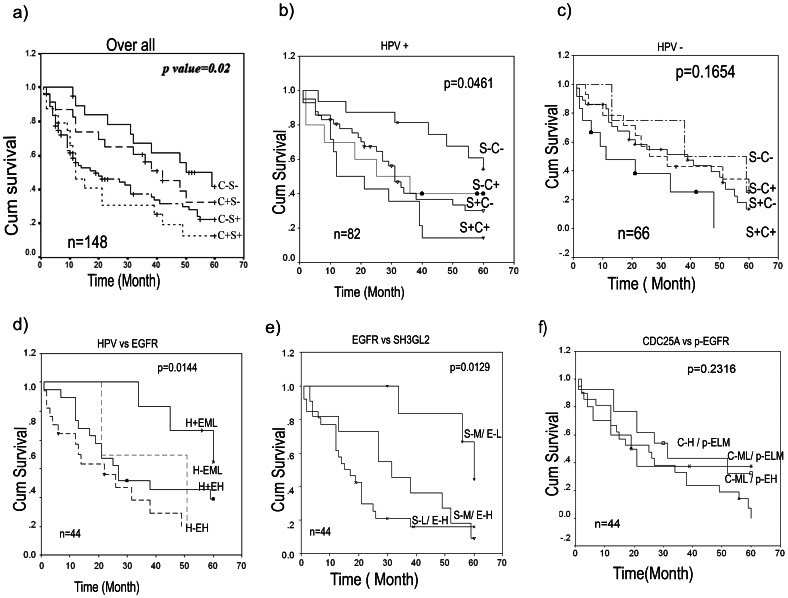
Kaplan–Meier analysis of survival (up to 5 years) of HNSCC patients. a) Co-alteration of CDC25A and SH3GL2 was significantly associated with poor overall survival (OS). b) The significant association with poor overall survival of patients having co-alterations of the genes was also seen in presence of HPV infection; however, co-alterations did not associate significantly with OS in absence of HPV infection (c). d) Poor survival was also seen of the patients having high EGFR expression irrespective of HPV infection. d) Similarly, reduced SH3GL2 expression and high EGFR expression was a predictor for poor OS. However f), protein expression of p-EGFR and CDC25A did not show any significant association with survival of the patients. Survival time was defined as the time from the date of surgery to the date of last follow-up, known recurrence or death (up to 5 years). n, total number of samples; C+/−, CDC25A deletion present/absent; S+/−, SH3GL2 alterations present/absent; H+/−, HPV infection present/absent; EH/ML, EGFR protein expression high/medium to low; S-L/MH, SH3GL2 protein expression low/medium to high; C-H/ML, CDC25A protein expression high/medium to low; p-EH/LM, p-EGFR protein expression high/low to medium.

Log rank test uncovered a statistically significant difference in overall survival between cases with and without alterations in *CDC25A* and *SH3GL2* (p = 0.02). The patients having alterations in both *SH3GL2* and *CDC25A* had the worse overall survival indicating prognostic significance of these genes among the HNSCC patients (Figure 7a). In presence of HPV infection, the patients having alterations in either or both *CDC25A* and *SH3GL2* genes showed poor survival (p = 0.0461) (Figure 7b) whereas, no such association has been seen in patients without HPV infection (Figure 7c). Interestingly, worse prognosis of the patients have been seen with i) high *EGFR* protein expression and absence of HPV infection (Figure 7d) and ii) with high *EGFR* and low *SH3GL2* protein expression in tumors (Figure 6e). On the contrary, no significant differences in survival of patients were observed having low or high protein expression of *CDC25A* and *p-EGFR* (Figure 7f).

In univariate analysis alterations of *SH3GL2*, *EGFR* amplification and absence of HPV infection showed hazardous to survival of the patient (Table 4). Similarly, in multivariate analysis alterations in *SH3GL2* (p, 0.0305; HR, 1.69; CI, 1.0508–2.7212) and *EGFR* amplification (p, 0.0036; HR, 1.905; CI, 1.234–2.938) in absence of HPV infection (p, 0.04760; HR, 0.7182; CI, 0.4695–1.098) were significant predictors for hazardous life and poor survival of patients (Table 4) were significant predictors for hazardous life and poor survival of patients (Table 4).

**Table 4:** Univariate and Multivariate analyses of genetic, clinical, and etiological parameters in predicting overall survival (OS) of 148 HNSCC patients.

Univariate	Over all survival		
Variable	p value	HR	95% CI for HR
SH3GL2	**0.0288**	1.6601	1.0539–2.61
CDC25A	0.502	1.1546	0.7589–1.7567
EGFR amp+	**0.0006**	2.0799	1.366–3.166
HPV+	**0.035**	0.6491	0.4336–0.9716
Grade	.2104	0.8002	0.5645–1.1342
Stage	.8976	0.9861	0.7970 1.2201
Node+	.6278	0.8940	0.5683 1.4063
Tobacco+	.5532	1.1530	0.7203 1.8456
Multivariate			
SH3GL2	**0.0305**	1.6910	1.0508–2.7212
CDC25A	0.5015	1.1622	0.7497–1.8016
EGFR amp+	**0.0036**	1.9050	1.2349–2.9386
HPV+	**0.0476**	0.7182	0.4695–1.0985
Grade	0.2510	0.8105	0.5662–1.1602
Stage	0.8349	1.0265	0.8025–1.3131
Node+	0.8289	0.9443	0.5616–1.5877
Tobacco+	0.7770	1.0746	0.6533–1.7677

Amp+, Gene amplification present.

## Discussion

The aim of the study is to understand the molecular mechanism of overexpression of *EGFR* protein in primary head and neck lesions. *EGFR* was found to be overexpressed in majority (66–84%) of dysplastic and HNSCC samples (Figure 2a) while, a low frequency of amplification (26.4%) was observed in these samples (Figure 2b). Low frequency of *EGFR* locus amplification (10–30%) has also been reported in HNSCC [4]. In contrast to the mutation in kinase domain of *EGFR* related to lung cancer, we did not find any such mutations. Like our data, infrequent mutation of *EGFR* has been reported in HNSCC [4]. However, mechanism of *EGFR* overexpression in this tumor having absence of genetic alterations in *EGFR* has not yet been elucidated. Although, a significant correlation was seen between gene amplification and mRNA expression, protein overexpression did not correlate with mRNA expression status of *EGFR* (Table 2). Similar to our study, a low frequency (<10%) of gene amplification and mutation have been reported in different cancers including HNSCC in Cancer Genome Project database [22]. This clearly suggests that expression of *EGFR* is not regulated transcriptionally and mechanism other than gene amplification/mutations might be responsible for observed overexpression of this protein in HNSCC tumors.

To understand the mechanism of *EGFR* protein overexpression we analyzed alterations (deletion/methylation/mutation/expression) of *SH3GL2* and *CDC25A* genes associated with *EGFR* homeostasis. Frequent deletion/methylation of *SH3GL2* (68%, 121/178) was evident in the head and neck lesions with comparable frequencies (63% to 77%) in dysplastic lesions and HNSCC samples (Figure S4) similar to that has been seen in our earlier study [1]. This has been suggested to be associated with the development of dysplastic lesions of this tumor. Frequent deletion (31–38%) and promoter methylation (34–36%) of *SH3GL2* has been reported in breast and lung carcinoma [23], [24] and also deletion in pituitary adenoma, neuroblastoma and pilocytic astrocytoma [25], [26], [27]. Similarly, in cancer genome project database, this gene has been reported to be inactivated by deletion in wide variety of human cancer [28] in varying frequencies. The alterations of *SH3GL2* could lead to its reduced expression as evident from the quantitative RT-PCR and IHC analysis. The reduced expression of *SH3GL2* was associated with alterations in this gene (Table 2). Similar to our data, its reduced expression was reported in HNSCC and carcinomas of breast and larynx [1], 10,23. Infrequent mutation of *SH3GL2* has been seen in the samples except two novel SNPs (rs112820965 & rs201266191) in the promoter region of this gene (Figure S2). The SNP (rs201266191) overlaps with the transcription factor (GCF) binding site and the minor allele (T) is prevalent in patients suggesting its importance in this tumor development. However, detailed study in this regard is warranted to understand the importance of this SNP in expression of this gene. Interestingly, alterations (deletion/promoter methylation) and reduced protein expression of *SH3GL2* showed significant association with *EGFR* protein overexpression in the head and neck lesions (Table 2). Moreover, the inverse expression pattern of these proteins in basal layer of normal oral epithelium indicates that down regulation of *SH3GL2* is needed for overexpression of *EGFR* (Figure 2a, Figure 4a).

*SH3GL2* mediated *EGFR* degradation has been validated in demethylation experiment by 5-aza-dc in Hep2 and siRNA mediated knock down of SH3GL2 in SCC084 cell lines (Figure 5). Increase in *SH3GL2* mRNA expression after 5-aza-dc treatment confirms the promoter methylation of the gene in Hep2 (Figure S5). Similarly in kinetics analysis, gradual increase in *SH3GL2* expression and gradual decrease in *EGFR* expression with increasing time of 5-aza-dc treatment clearly suggest the association of *SH3GL2* with *EGFR* homeostasis as evident by western blot analysis (Figure 5a &b). This was further confirmed by ICC analysis after 5-aza-dc treatment where a decrease in *EGFR* protein expression and increase in *SH3GL2* expression were markedly visualized (Figure 6). The association of SH3GL2 and EGFR was also confirmed by siRNA mediated knock down of SH3GL2 in SCC084 cell line (Figure 5c &d). The upregulation of EGFR, as seen in our study, has also been reported by Shang et al in Hep2 cell line [29]. Similarly, Dasgupta et al reported that upregulation of SH3GL2 expression could induce EGFR protein internalization and degradation in lung cancer cell lines [24]. This suggests that inactivation of *SH3GL2* might impair *EGFR* endocytosis for degradation resulting its stabilization in HNSCC.

To find out the association of *CDC25A* with EGFR phosphorylation in HNSCC, molecular alterations (deletion/methylation/expression) of *CDC25A* and expression of *p-EGFR* were done in same set of the head and neck lesions. Frequent deletion of *CDC25A* was seen in dysplastic lesions and subsequent stages as seen our previous report (Figure 4b, f) [14]. Deletion of *CDC25A* has also been reported (cancer genome project database) in varying frequencies in numerous human cancer [30]. Also, high frequency of reduced expression (RNA/protein) of *CDC25A* was seen in HNSCC samples as seen in our earlier study [14]. Differential expression (RNA/protein) pattern of *CDC25A* (30–80%) has been reported in different carcinomas in liver, esophagus, colon, breast including head and neck [31]. But none has analyzed the alterations (deletion/methylation/mutation) of this gene in these tumors. Though no methylation of *CDC25A* was observed in our samples, the upregulation of this gene by 5-aza-dc in Hep2 cell line suggests the presence of methylation in the some other regulatory regions (Figure 5 & Figure 6). Further study in this regard is needed to identify the regulatory regions of this gene. The significant association of reduced *CDC25A* expression with overexpression of p*-EGFR* in this tumor suggests their synergistic action in development of tumor (Table 2). This was validated in Hep2 cell line after 5-aza-dc treatment where upregulation of CDC25A and down regulation of p-EGFR were evident (Figure 6). On the other hand, EGFR/p-EGFR level was upregulated due to knock down CDC25A by siRNA treatment (Figure 5 c & d). Similarly, the inverse expression pattern of *p-EGFR* and *CDC25A* was evident in the basal layer of normal oral epithelium (Figure 4b, c). To the best of our knowledge, this is the first report of regulation of *EGFR* phosphorylation by CDC25A in head and neck lesions.

The worse prognosis of the patients having low *SH3GL2* and high *EGFR* expression (Figure 7e) suggests their prognostic importance. Similarly, co-alterations of *SH3GL2* and *CDC25A* in tumors could also be used as prognostic marker for poor outcome of patients (Figure 7a). Similar trend has also been seen in HPV infected patients (Figure 7b). It was evident that HPV viral oncoprotein E5 (expressed from episomal form of the virus) could inhibit the EGFR endocytosis by disrupting the c-Cbl–EGFR interaction [32]. But in invasive tumor, HPV is mainly in integrated form with loss of E5 expression. Thus, association between HPV and EGFR expression in invasive tumor, if any, should be analyzed in detail. On the other hand, hazardous life was predicted for the patients having EGFR amplification, alterations of SH3GL2 and absence of HPV infection in tumors (Table 4).

Thus, overexpression of *EGFR*/*p-EGFR* is due to impairment of its homeostasis mechanism in HNSCC. The detailed analysis of EGFR homeostasis pathway in HNSCC is warranted to develop proper therapeutic measure of the tumor.

## Supporting Information

Figure S1 **Molecular alterations of EGFR.**
(TIF)

Figure S2 **Molecular alterations of SH3GL2.**
(TIF)

Figure S3 **ICC analysis of EGFR, p-EGFR, SH3GL2 and CDC25A in presence and absence of 5-aza-dc.**
(TIF)

Figure S4 **Alterations(deletion/methylation) pattern of SH3GL2 and CDC25A in dysplasia and HNSCC samples.**
(TIF)

Figure S5 **Demethylation experiment of SH3GL2 and CDC25A in Hep2 cell line.**
(TIF)

Table S1 **Clinical information of control samples.**
(DOC)

Table S2 **Primer profile used in different experiment.**
(DOC)

Table S3 **Information of microsatellite markers.**
(DOC)

Table S4 **Correlation between DPCR and QPCR.**
(DOC)

Table S5 **Molecular alterations of EGFR.**
(DOC)

Table S6 **Association of alterations of genes with different clinicopathological parametres.**
(DOC)

Table S7 **Compilation of mutation analysis of SH3GL2.**
(DOC)

Table S8 **Effect of 5-aza-dc treatment on expression of EGFR, SH3GL2 and CDC25A.**
(DOC)

Doc S1 **Supplementary methods.**
(DOC)
