# Supplementary figures and images for: Overexpression of EGFR in Head and Neck Squamous Cell Carcinoma Is Associated with Inactivation of SH3GL2 and CDC25A Genes
Source: PLoS One. 2013 May 10;8(5):e63440. doi: 10.1371/journal.pone.0063440 (PMC3651136; doi:10.1371/journal.pone.0063440)

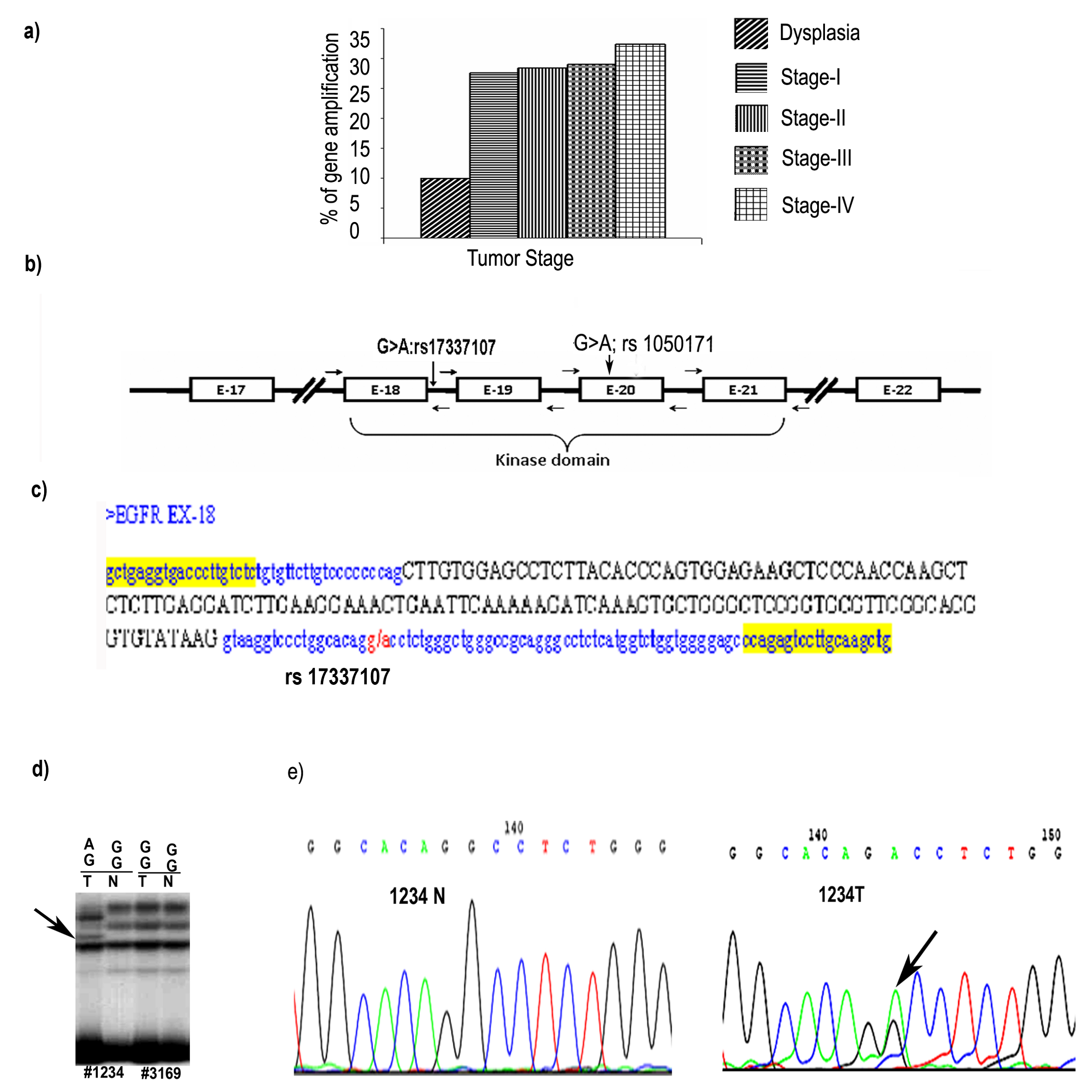

Supplement: Figure S1 — Molecular alterations of EGFR. (TIF) [file pone.0063440.s001.tif]

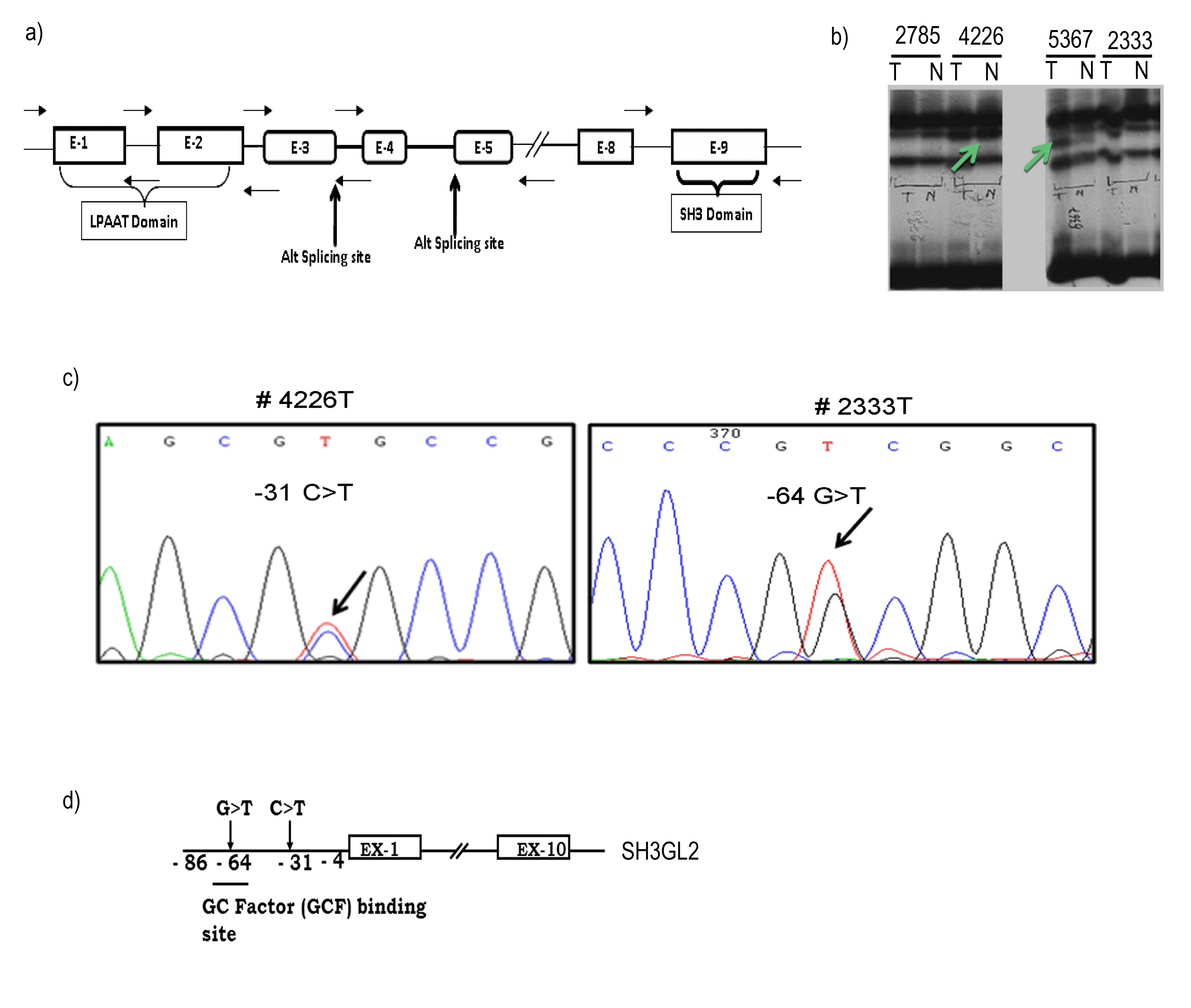

Supplement: Figure S2 — Molecular alterations of SH3GL2. (TIF) [file pone.0063440.s002.tif]

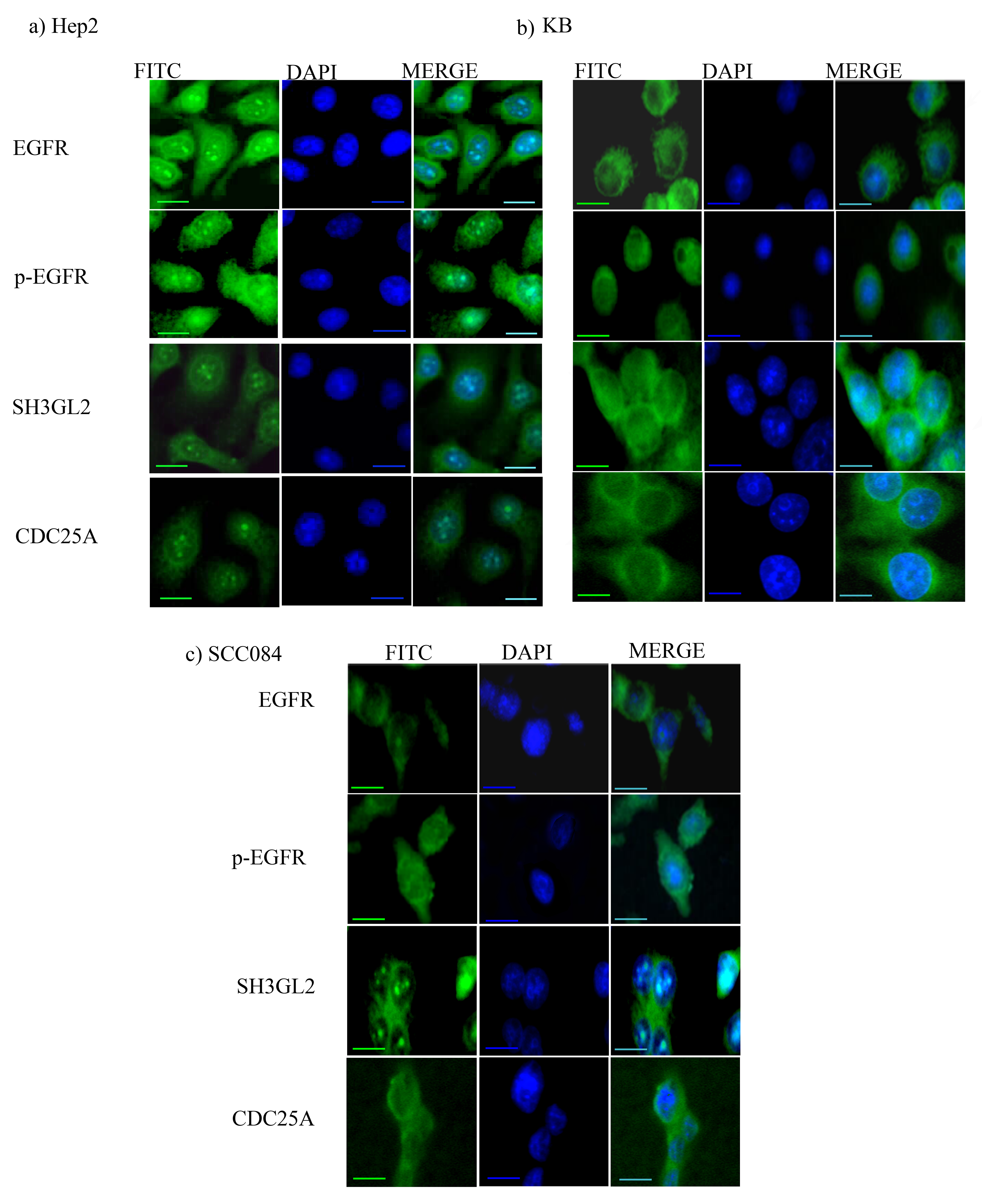

Supplement: Figure S3 — ICC analysis of EGFR, p-EGFR, SH3GL2 and CDC25A in presence and absence of 5-aza-dc. (TIF) [file pone.0063440.s003.tif]
