# Supplementary material for: Overexpression of EGFR in Head and Neck Squamous Cell Carcinoma Is Associated with Inactivation of SH3GL2 and CDC25A Genes
Source: PLoS One. 2013 May 10;8(5):e63440. doi: 10.1371/journal.pone.0063440 (PMC3651136; doi:10.1371/journal.pone.0063440)
Supplement: Table S1 — Clinical information of control samples. (DOC) [file pone.0063440.s006.doc]

Table S1. Information of unrelated control

| Control Subject (52) |  | Male, N (%) | 43 (82.7%) |
| --- | --- | --- | --- |
|  |  | Female, N(%) | 9 (17.3%) |
|  | Mean Age |  | 42.4±6.7 |
