# Supplementary material for: Overexpression of EGFR in Head and Neck Squamous Cell Carcinoma Is Associated with Inactivation of SH3GL2 and CDC25A Genes
Source: PLoS One. 2013 May 10;8(5):e63440. doi: 10.1371/journal.pone.0063440 (PMC3651136; doi:10.1371/journal.pone.0063440)
Supplement: Table S2 — Primer profile used in different experiment. (DOC) [file pone.0063440.s007.doc]

| **Primers** | **Location** | **Analytical purpose** | **Sense** | **Antisense** | **Size (bp)** |
| --- | --- | --- | --- | --- | --- |
| **EGFREX-18** | Flanking exon 18 | Mutation analysis | 5-GGT GAC CCT TGT CTC TGT GT-3’ | 5-TAT ACA GCT TGC AAG GAC TCT G-3 | 243 |
| **EGFREX-19** | Flanking exon 19 | Mutation analysis | 5-GTG CAT CGC TGG TAA CAT CCA-3 | 5- CTG AGG TTC AGA GCC ATG GA 3’ | 240 |
| **EGFREX-20** | Flanking exon 20 | Mutation analysis | 5’-TCT GGC CAC CAT GCG AAG C-3’ | 5’GGATCCTGGCTCCTTATCTC-3 | 283 |
| **EGFREX-21** | Flanking exon 21 | Mutation analysis | 5’AGAGCTTCTTCCCATGATGATC-3 | 5- TGGTCCCTGGTGTCAGGA-3 | 260 |
| **EGFR-RT** | Coding region of exon 15& 16 | mRNA expression | 5-CCA GAC AAC TGT ATC CAG TG-3 | 5-GTT GGA CAG CCT TCA AGA C-3 | 189 |
| **DDR2** | Intragenic region | control for gene amplification | 5’-GATGATGATCTGGAGAGGCAGAAC-3’ | 5’-GAAGACGATGACAGCGATGAG-3’ | 129 |
| **SH3GL2 Ex-1** | Flanking exon 1 | Methylation & Mutation | 5' GAGTGTTTCTCCGCAAGAGC 3' | 5' TGAGTGGCTTTATGGAACTGC 3' | 217 |
| **SH3GL2 EX 2** | Flanking exon 2 | Mutation analysis | 5-AGACTCTCCACCTAAGTCA-3 | 5-ATGTCAAAGGGAACACTAGG-3 | 238 |
| **SH3GL2 Ex.3** | Flanking exon 3 | Mutation analysis | 5’- CCGGTATTCTAAAGCTCATC-3’ | 5’- AAGTCGCTAACCTCTCAGAA-3’ | 266 |
| **SH3GL2 Ex4-5** | Flanking exon 4 & 5 | Mutation analysis | 5’- GTGTCACATTGCCTACTCTG-3’ | 5’- TGTGAGGAAGCACTTGTTAC-3’ | 210 |
| **SH3GL2 Ex. 10** | Flanking exon 10 | Mutation analysis | 5' CAGGCAGCAGATTCTGTGAG 3' | 5' GGCAACCAGAATTTCCACAT 3' | 263 |
| **SH3GL2 RT** | Coding region of exon 9 &10 | mRNA expression | 5' CACGAATGAGCCTGG 3' | 5' GGCAACCAGAATTTCCACAT 3' | 272 |
| **D9S157** | intragenic of SH3GL2 | Deletion analysis | 5' CATTTCATCTGGTAGACCCA 3' | 5' TTTGATTGGCTGGAAGTAGA 3' | 217-239 |
| **SH3GL2(M)** | Promoter region | For MSP analysis | 5'-GTATTTGTTTGGGCGTTTTC-3' | 5'-CCTAACCGCCTCTAATCGTA-3' | 171 |
| **SH3GL2(U)** | Promoter region | For MSP analysis | 5'-AGTATTTGTTTGGGTGTTTTT-3' | 5'-TCCCCTAACCACCTCTAATCA-3' | 175 |
| ***CDC25A* meth** | -178 to +91 bp ^#^ | Methylation | 5’-GAAGTTGCTTAC TGATTGGTG-3’ | 5’-GTATAAATCCAAACAAACGTG-3’ | 269 |
| **D3S3560** | Intragenic of CDC25A | Deletion analysis | 5'-CCTTATGCCCTTTGTCAAGA-3' | 5'-TGCAGTTATGTATGAGAACATCCT-3' | 179-183 |
| ***CDC25A RT*** | Coding region of exons | mRNA expression | 5’-GCCATTGGACAGTAAAGAAA-3’ | 5’-CATCATCCTCATCAGACAAA-3’ | 260 |
| ***RARβ2* ( K2)** | Coding region of exon 1 | DNA integrity control | 5’-AGAGTTTGATGGAGTTGGGT-3’ | 5’-CATTCGGTTTGGGTCAATCC-3’ | 229 |
| ***β-3A ADAPTIN* (K1)** | Exon 1-intron1 | DNA digestion control | 5’-TGCCCTCTGGACTGGAACCT-3’ | 5’-CCTGAGCCCAGCCCAAGTC-3’ | 445 |
| ***B2M*** | Coding region of exon | Control for expression | 5’-GTGCTCGCGCTACTCTCTCT-3’ | 5’-TCAATGTCGGATGGATGAAA-3’ | 143 |
|  |  |  |  |  |  |

Table S2. Details of the oligonucleotide Primer of the genes used in different experiment
