# Supplementary material for: Overexpression of EGFR in Head and Neck Squamous Cell Carcinoma Is Associated with Inactivation of SH3GL2 and CDC25A Genes
Source: PLoS One. 2013 May 10;8(5):e63440. doi: 10.1371/journal.pone.0063440 (PMC3651136; doi:10.1371/journal.pone.0063440)
Supplement: Table S3 — Information of microsatellite markers. (DOC) [file pone.0063440.s008.doc]

| **Marker** | **Dist. From p-ter (Mb)** | **Type of repeat** | **Ann. Temperature** | **Conc. Of Mgcl2** |
| --- | --- | --- | --- | --- |
| D3S3560 | 48.16 at chr. 3 | (CA) n | 52°C | 2.5mM |
| D9S157 | 17.61 at chr. 9 | (TG) n | 55^0^C | 1.5mM |

Table S3. Microsatellite marker profile of D3S3560 and D9S157
