# Supplementary material for: Overexpression of EGFR in Head and Neck Squamous Cell Carcinoma Is Associated with Inactivation of SH3GL2 and CDC25A Genes
Source: PLoS One. 2013 May 10;8(5):e63440. doi: 10.1371/journal.pone.0063440 (PMC3651136; doi:10.1371/journal.pone.0063440)
Supplement: Table S4 — Correlation between DPCR and QPCR. (DOC) [file pone.0063440.s009.doc]

Table S4. Correlation between the gene amplification analysis using differential polymerase chain reaction (DPCR) and quantitative polymerase chain reaction (QPCR) of EGFR.

| Sample | DPCR | QPCR |
| --- | --- | --- |
| L50 | A+ | + |
| 3371 | A+ | + |
| 4892 | A- | - |
| 5232 | A- | - |
| 3484 | A- | - |
| 3689 | A- | - |
| 6392 | A+ | - |
| 6817 | A+ | + |
| 2507 | A- | + |
| 4119 | A- | - |
| 1087 | A+ | + |
| 2888 | A+ | + |
| 219 | A+ | + |
| 5671 | A- | - |
| 2123 | A+ | + |
| 1234 | A- | + |
| 914 | A- | - |
| 944 | A+ | + |
| 4226 | A- | - |
| 6814 | A+ | + |
| % of A+ | 11/20 | 13/20 |
| p value= | 0.0007* | |

A+/-; amplification present/ absent, * statistically significant
