# Supplementary material for: Overexpression of EGFR in Head and Neck Squamous Cell Carcinoma Is Associated with Inactivation of SH3GL2 and CDC25A Genes
Source: PLoS One. 2013 May 10;8(5):e63440. doi: 10.1371/journal.pone.0063440 (PMC3651136; doi:10.1371/journal.pone.0063440)
Supplement: Table S5 — Molecular alterations of EGFR. (DOC) [file pone.0063440.s010.doc]

**Table S5. a) Mutation analysis of different exons of EGFR.**

| Exons of EGFR | Frequency of abnormal band | Type of nucleotide change | Frequency of mutation |
| --- | --- | --- | --- |
| **Exon-18** | 13% (24/178) | **G > A SNP** | **7%(12/178)** |
| **Exon-19** | **0%(0/178)** | **-** | **-** |
| **Exon-20** | 20% (35/178) | **G > A SNP** | **-** |
| **Exon-21** | **0%(0/178)** | **-** | **-** |

**Table S5. b) Distribution of rs17337107 G>A Variant in HNSCC patient.**

| **No. of sample= 16** | **Genotype** | **DNA samples** | | **P value** |
| --- | --- | --- | --- | --- |
|  |  | **Tumour (24)** | **Normal (24)** |  |
|  | **Genotype GG** | 12 | 0 |  |
|  | **Genotype AG** | 12 | 24 |  |
|  | **Genotype AA** | 0 | 0 |  |
|  | **Allele A** | 12 | 24 | 0.0203 |
|  | **Allele G** | 36 | 24 |  |

**Table S5. c) Distribution of rs1050171G>A Variant in HNSCC patient.**

| **No. of sample= 24** | **Genotype** | **DNA samples** | |
| --- | --- | --- | --- |
|  |  | **Tumour (35)** | **Normal (35)** |
|  | **Genotype GG** | 13 | 13 |
|  | **Genotype AG** | 14 | 14 |
|  | **Genotype AA** | 8 | 8 |
|  | **Allele A** | 30 | 30 |
|  | **Allele G** | 40 | 40 |
