# Supplementary material for: Overexpression of EGFR in Head and Neck Squamous Cell Carcinoma Is Associated with Inactivation of SH3GL2 and CDC25A Genes
Source: PLoS One. 2013 May 10;8(5):e63440. doi: 10.1371/journal.pone.0063440 (PMC3651136; doi:10.1371/journal.pone.0063440)
Supplement: Table S6 — Association of alterations of genes with different clinicopathological parametres. (DOC) [file pone.0063440.s011.doc]

| Clinical | SH3GL2 | | Total | Overall | CDC25A | | Total | Overall |
| --- | --- | --- | --- | --- | --- | --- | --- | --- |
| parameter | Alt+ | Alt- |  | p value | ALT+ | Alt- |  | p value |
| Dysplasia | 19 | 22 | 30 |  | 6 | 24 | 30 |  |
| Stage I | 14 | 4 | 18 | **0.02** | 4 | 14 | 18 | **0.23** |
| Stage II | 28 | 14 | 42 |  | 11 | 31 | 42 |  |
| Stage III | 31 | 16 | 47 |  | 19 | 29 | 48 |  |
| Stage IV | 30 | 10 | 40 |  | 16 | 24 | 40 |  |
| Lymph node + | 29 | 15 | 44 | **0.66** | 19 | 25 | 44 | **0.65** |
| Lymph node - | 74 | 30 | 104 |  | 37 | 67 | 104 |  |
| Tobacco+ | 89 | 37 | 126 | **0.31** | 42 | 84 | 126 | **0.34** |
| Tobacco- | 32 | 20 | 52 |  | 14 | 38 | 52 |  |
| HPV+ | 63 | 30 | 93 | **0.92** | 33 | 60 | 93 | **0.37** |
| HPV- | 58 | 27 | 85 |  | 23 | 62 | 85 |  |
| EGFR amp+ | **32** | **15** | **47** | **0.86** | 19 | 28 | **47** | **0.62** |
| EGFR amp - | 90 | 41 | 131 |  | 37 | 94 | 131 |  |
| SH3GL2 ALT+ | x | x | x | x | 32 | 89 | 121 | **0.05** |
| SH3GL2 ALT- | x | x | x |  | 24 | 33 | 57 |  |
| CDC25A Alt+ | 32 | 24 | 56 | **0.05** | x | x | x | x |
| CDC25A Alt- | 89 | 33 | 122 |  | x | x | x |  |

Table S6. Correlation of alterations of SH3GL2 and CDC25A with different clinical parameter
