# Supplementary material for: Overexpression of EGFR in Head and Neck Squamous Cell Carcinoma Is Associated with Inactivation of SH3GL2 and CDC25A Genes
Source: PLoS One. 2013 May 10;8(5):e63440. doi: 10.1371/journal.pone.0063440 (PMC3651136; doi:10.1371/journal.pone.0063440)
Supplement: Table S7 — Compilation of mutation analysis of SH3GL2. (DOC) [file pone.0063440.s012.doc]

| **No. of samples** =  22 tumor pair + 52 disease free samples | Genotype | DNA samples | | |
| --- | --- | --- | --- | --- |
|  |  | Tumor(32) | Normal(32) | Control(52) |
|  | CC | 30 | 30 | 50 |
|  | CT | 2 | 2 | 2 |
|  | TT | 0 | 0 | 0 |
|  | Allele C | 62 | 62 | 102 |
|  | Allele T | 2 | 2 | 2 |
|  | C allele frequency | 0.97 | 0.97 | 0.98 |
|  | T allele frequency | 0.03 | 0.03 | 0.02 |

**Table A.7 a) Compilation of the mutations of SH3GL2 in head and neck lesions.**

| Exons of SH3GL2 | Frequency of abnormal band shift | Type of nucleotide change | Frequency of mutation |
| --- | --- | --- | --- |
| Exon-1 | 16% (32/178) | SNP | - |
| Exon-2 | 0% (0/178) | - | - |
| Exon-3 | 0% (0/178) | - | - |
| Exon-4-5 | 0% (0/178) | - | - |
| Exon-10 | 0% (0/178) | - | - |

**Table S7. b) Distribution of *-*31 C>T (**rs112820965) **nucleotide variation in SH3GL2 gene in HNSCC patients and control samples.**

**Table S7. c) Distribution of -64 G>T nucleotide variation (**rs201266191**) in SH3GL2 gene in HNSCC patients and control samples.**

| **No. of samples** =  22 tumor pair + 52 disease free samples | Genotype | DNA samples | | |
| --- | --- | --- | --- | --- |
|  |  | Tumor(32) | Normal(32) | Control(52) |
|  | GG | 22 | 22 | 48 |
|  | GT | 10 | 10 | 4 |
|  | TT | 0 | 0 | 0 |
|  | Allele G | 54 | 54 | 100 |
|  | Allele T | 10 | 10 | 4 |
|  | G allele frequency | 0.84 | 0.84 | 0.96 |
|  | T allele frequency | 0.16 | 0.16 | 0.04 |
