# Supplementary material for: Overexpression of EGFR in Head and Neck Squamous Cell Carcinoma Is Associated with Inactivation of SH3GL2 and CDC25A Genes
Source: PLoS One. 2013 May 10;8(5):e63440. doi: 10.1371/journal.pone.0063440 (PMC3651136; doi:10.1371/journal.pone.0063440)
Supplement: Table S8 — Effect of 5-aza-dc treatment on expression of EGFR, SH3GL2 and CDC25A. (DOC) [file pone.0063440.s013.doc]

Table S8. Pecrent of expression change of EGFR, SH3GL2 and CDC25A after 5-aza-dc treatment at different time points.

| Time of aza treatmet (hour) | % of decrease in EGFR expression | % of increase in SH3GL2 expression | % of increase in CDC25A expression |
| --- | --- | --- | --- |
| 0 | 0 | 0 | 0 |
| 24 | 10.47 | 165.10 | 55.63 |
| 48 | 40.13 | 222.65 | 116.61 |
| 72 | 61.42 | 959.30 | 144.78 |
| 96 | 79.03 | 1165.67 | 206.91 |
| 120 | 90.50 | 1542.79 | 261.91 |
