# Supplementary material for: Overexpression of EGFR in Head and Neck Squamous Cell Carcinoma Is Associated with Inactivation of SH3GL2 and CDC25A Genes
Source: PLoS One. 2013 May 10;8(5):e63440. doi: 10.1371/journal.pone.0063440 (PMC3651136; doi:10.1371/journal.pone.0063440)
Supplement: Doc S1 — Supplementary methods. (DOC) [file pone.0063440.s014.doc]

**Supplimentary Material and Methodes**

**Gene amplification analysis of EGFR:**

The primers of both EGFR and DRD2 were co-amplified by PCR. The PCR products were visualized after electrophoresis in 2% agarose gel followed by ethidium bromide staining. The band intensities were measured by densitometry scanning. EGFR amplification level in tumor DNA (T) was calculated as follows:

Relative copy number

=

T_EGFR_

/ T_DDR2_

N_EGFR_/N_DDR2_

Where T_EGFR_ and T _DDR2_ are the intensities of the bands in tumor DNA samples and N_EGFR_ and N_DDR2_ are the intensities of the bands in corresponding normal Samples. EGFR locus was considered as amplified when the ratio will be ≥2 (Bhattacharya et al, 2009 ).

The amplification of the gene was validated by real time PCR using 2x SYBR Green master mix according to manufacturer (Applied Biosystem, USA ) (Ghosh et al, 2009 ). Briefly, we quantified each gene (EGFR and DDR2) in separate reactions. Q-PCR reactions for each sample and each gene were performed in triplicate. The threshold amplification cycles (CT) at the normalized reporter signal minus the baseline signal level of 0.2 for each gene were determined, and their differences CT (DDR2 - EGFR) were calculated. The cutoff for normal EGFR copy number was determined in DNA of normal WBCs from 30 healthy volunteers. The EGFR copy number was considered increased if the value was > 2; normal if the value was more than 1.5 but less than 2, and decreased if the value was < 1.5.

**Western Blot analysis**

Proteins were extracted from nearly 10^7^ cells grown to 90% confluency. For whole cell extract (WCE) cells were homogenized in a Teflon homogenizer with RIPA buffer (25mM Tris-HCl pH 7.6, 1mM EDTA pH 8.0, 150mM sodium chloride, 1% NP-40, 1% sodium deoxycholate, 0.1% SDS, 1mM PMSF, 1-2µg/µl leupeptin and 1-2µg/µl aprotinin). The homogenate was centrifuged at 12,000 rpm for 15 minutes at 4^o^C and the supernatant was quantified, aliquoted and stored at -80^o^C . About100 µg were loaded on 10 % SDS-polyacrylamide gel with a 4% stacking gel according to standard procedure. Separated proteins were transferred to Immobilion P-polyvinylidine difluoride membrane (PVDF: Millipore, MA). The membrane was blocked in Tris-buffered saline/0.05% Tween20 (TBS-T) with 5% NFDM for 1 hour at room temperature and incubated with primary antibody for EGFR at 1:1000 dilution at 4^o^C for overnight. The membrane was then washed thrice with TBS-T (0.1% Tween20) and further incubated with secondary antibody at a dilution of 1:5,000 at room temperature for 1 hour. After washing thrice membrane was developed with luminol and the signal intensities were scanned by densitometric scanning (Bio-Rad GS-800, USA). The membrane was then stripped using 0.2M NaOH as stripping buffer and subsequently incubated with primary antibodies for SH3GL2, CDC25A and tubulin and corresponding secondary antibodies, all procured from Santa Cruz Biotechnology, CA, USA.

**Promoter methylation:**

The methylation status of SH3GL2 and CDC25A promoter was assessed by PCR-based methylation sensitive restriction analysis (MSRA) according to the protocol of Loginov et al. (2004) using methylation sensitive enzyme HpaII in 30 dysplastic lesions and 148 HNSCC samples. The 445-bp fragment of β-3A adaptin gene (K1) and 229-bp fragment of RARb2 exon-1 (K2) was used as digestion and integrity controls, respectively. To validate the methylation data obtained by MSRA, methylation-specific PCR (MSP) in 30 primary HNSCC samples and 3 oral cancer cell lines were done after bisulfite modification of the DNA according to standerd procedure.

Briefly, 2 μg of genomic DNA was denatured with 0.2M NaOH for 15min at 37°C. Cytosines were sulfonated in the presence of 3M sodium-bisulfite (Sigma Chemical Co., USA) and 10mM hydroquinone (Sigma Chemical Co., USA) for 16hr at 55°C. Thereafter, the DNA samples were desalted using Wizard DNA Clean-Up System (Promega, Madison, WI, USA) and desulfonated in 0.3M NaOH at 37°C for 15 min. Finally, the treated DNA samples were precipitated with ethanol and resuspended in TE buffer. The PCR of the modified DNA was done with methylated specific/ unmethylated specific (M/U) primers sets (Table S2) according to the standard procedure (Herman et al. 1996). PCR products were analyzed on 2% agarose gels, visualized under UV illumination and photographed. PCR products were analyzed on 2% agarose gel, visualized under UV illumination and photographed. Combining the methylation status obtained from two methods, final score of methylation frequency was calculated.
